# Dataset for multimodal transport analytics of smartphone users - Collecty

**DOI:** 10.1016/j.dib.2023.109481

**Published:** 2023-08-06

**Authors:** Martina Erdelić, Tomislav Erdelić, Tonči Carić

**Affiliations:** University of Zagreb, Faculty of Transport and Traffic Sciences, Department of Intelligent Transportation Systems, Vukelićeva 4, 10000 Zagreb Croatia

**Keywords:** Transport dataset, User trajectories, Urban mobility analysis, Transport mode classification, Smartphone sensor data, Android application

## Abstract

Urban mobility is facing many challenges, such as energy consumption, pollution, and safety. Therefore, it is necessary to analyze the mobility of users through the transportation network using data containing information regarding the used transport mode. This data article describes a dataset from mobile devices collected by users as they move through the transportation network. Each sample in this dataset is labelled with a corresponding transport mode. Eight transport modes are present in the dataset: Car, Bus, Walking, Bicycle, Train, Tram, Running and Electric Scooter. The basic breakdown of the raw data according to users, transport modes and multimodal routes is presented. During data collection, data from the accelerometer, magnetometer, and gyroscope sensors mounted within the mobile device were stored. The data were collected using a mobile application from mobile devices with an embedded Android operating system. The structure of the text files in which the data were stored and the structure of the application used to collect the data are presented in the paper. The collected data provides a highly relevant basis for mobility analysis and planning, analysis of road conditions, clustering of user behaviour, and comparison of transport mode classification methods.

**Specifications Table**

**Table:** 

Subject	Data Science, Engineering
Specific subject area	Transportation analysis, applied machine learning, data engineering
Type of data	Table (.txt)
How the data were acquired	Data were acquired using a mobile application Collecty from mobile devices with an embedded Android operating system. Accelerometer, gyroscope, and magnetometer sensor data were collected from each mobile device.
Data format	Raw anonymized data.
Description of data collection	A sample of 15 individuals, representing a range of age groups, participated in a data collection spanning a duration of 5 months. Participants were instructed to activate the mobile application, which utilizes sensor data from the device, and to specify the transport mode being used. Upon reaching their destination, each participant was required to validate their route via the displayed digital map. The data collected pertained to transportation networks within Croatia.
Data source location	• City: City of Zagreb • Country: Croatia
Data accessibility	Repository name: Kaggle Data identification number: 10.34740/kaggle/dsv/4976711 Direct URL to data: Collecty dataset Kaggle The data can be downloaded upon registering.

## 1 Value of the Data

• The dataset increases the diversity of data in the area of multimodal transport activities and thus enables the improvement of existing methods for the classification of transport modes.
• Researchers in the field of Human Activity Recognition (HAR) and transport mode classification can compare existing and newly developed methods.
• The dataset contains raw sensor data for the e-scooter transport mode, which is not included in published datasets.
• The dataset can be used to extend the benchmark problem to additionally test spatial dependencies of transport mode classifiers.
• The dataset can be reused for analysis of sensor-based multimodal travel activities, trajectory segmentation, mobility analysis and planning, analysis of road conditions or clustering of user behaviour.

## 2 Objective

The current dataset is created to establish a benchmark for comparing transport mode classification methods. Additionally, it aims to enable spatial independence comparisons between different methods by utilizing more datasets collected from distinct transport networks. A dataset marked with transport modes is important in conducting a thorough mobility analysis, which can provide insights into different transport modes, enabling transportation network improvement with a focus on sustainable transport modes.

## 3 Data Description

The raw sensor data is organized within text files, with each file being designated a unique name in the format of UserID_TrajectoryID. The dataset comprises 454 text files, representing 454 distinct trajectories. Each trajectory is marked with a flag indicating user confirmation of the route. Only routes that have been confirmed by the user are included in the published dataset. Each text file comprises 18 columns of data, as detailed in Table 1 which includes the attributes and respective units of measurement. All timestamps are recorded in the 12-hour format (day/month/year hour:minute:second.millisecond). Each record includes multiple timestamps, with the first timestamp (line 1 in Table 1) corresponding to the time of sampling. Additional timestamps indicate the last change in sensor value prior to sampling, and these timestamps may not always be identical since the trigger for data collection is the timer tick rather than a change in the sensor measurement. The data is sampled at intervals of 10 milliseconds, resulting in a sampling frequency of 100 Hz. This specific sampling frequency is chosen to facilitate effective comparison with models developed using related datasets. For instance, the Sussex-Huawei Locomotion (SHL) dataset, which shares similarities with our dataset, also has a sampling frequency of 100 Hz [1]. Also, as a relatively high sampling frequency was used, other researchers could easily down-sample the data to facilitate their particular needs. Dataset labels are transport modes: Walk, Run, Bike, Car, Bus, Tramway, Train, E scooter and some data are labelled as *Unknown*. Samples are assigned the label *Unknown* when the transition between two transport modes takes too long. Out of 83227415 records in the dataset 37 of them had the label of the transport mode *Unknown*. These records are considered unreliable and are typically excluded from most research analyses. Additionally, records that lacked any recorded sensor measurements were entirely removed and are not included in the published dataset. It is important to note that all remaining data are presented in their raw form, without applying any filtering methods to address potential sensor errors in the measurements to accurately represent real-time conditions and ensure the authenticity of transport mode detection. This approach is crucial when developing a real-time model for transport mode detection, as it allows simulation of data arrival and simultaneous identification of the transport mode.

**Table 1:** Header of the raw sensor data text files.

No.	Attribute name	Measuring unit
1	Time	Date and time
2	Linear acceleration x axis	$m/s^{2}$
3	Linear acceleration y axis	$m/s^{2}$
4	Linear acceleration z axis	$m/s^{2}$
5	The time of the last acceleration change in the sample	Date and time
6	Rotation rate along the x axis	rad/s
7	Rotation rate along the y axis	rad/s
8	Rotation rate along the z axis	rad/s
9	The time of the last gyroscope change in the sample	Date and time
10	Gravitational acceleration along the x axis	$m/s^{2}$
11	Gravitational acceleration along the y axis	$m/s^{2}$
12	Gravitational acceleration along the z axis	$m/s^{2}$
13	The time of the last change in the gravity in the sample	Date and time
14	Magnetic field strength along the x axis	μT
15	Magnetic field strength along the y axis	μT
16	Magnetic field strength along the z axis	μT
17	The time of the last magnetometer change in the sample	Date and time
18	Transport mode	Walk, Run, Bike, Car, Bus, Tramway, Train, E scooter, Unknown

**Fig. 1:**
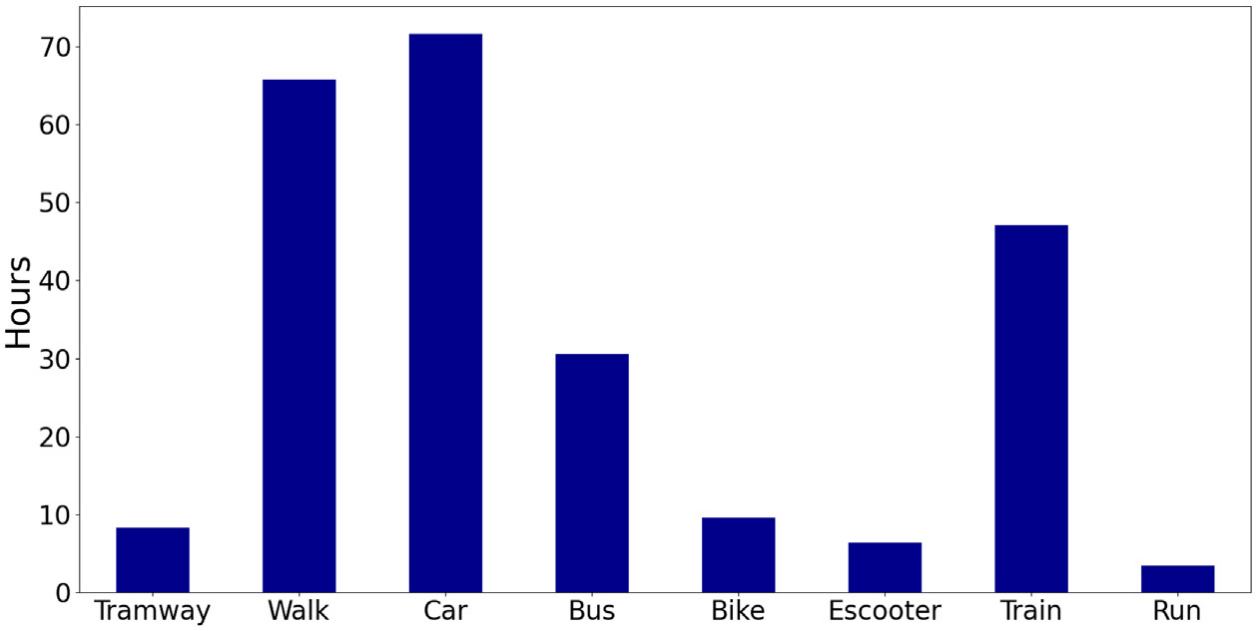
Distribution of data volume by transport mode.

The dataset was collected during a 5-month period, spanning from November 2021 to March 2022. The quantity of data collected for various transport modes is not uniform, as illustrated in Fig. 1. Considered transport modes are shown on the x axis and the hours of data collected on the y axis. The amount of data collected for the transport modes car, walking, train, and bus is higher than the amount of data collected for running, electric scooter, tram and bicycle. In total, approximately 242 hours of data were collected for all transport modes.

A more detailed distribution of data can be found in Table 2, which shows the amount of data per user for each transport mode in hours. For the data collection process, a group of 15 users who voluntarily registered was selected. The primary focus of the selection process was to ensure a diverse range of transport modes utilized by the participants, aiming to maximize the heterogeneity of the collected data. The focus was primarily on the variety of transport modes rather than specific user characteristics. It is evident that the amount of data collected varies among users. For instance, the dataset includes a higher amount of data from users with IDs 29, 23, and 18, compared to a significantly smaller amount of data from users with IDs 28, 26, and 20. For the majority of transport modes, data is represented from multiple users, while bicycling and electric scooters are represented by data from only two users. The low representation of these transport modes in user trajectories may be attributed to the dataset being collected during the winter months.

**Table 2:** Distribution of data by transport mode per user expressed in hours.

User ID	Walk	Run	Bike	Car	Bus	Train	Tram	E-scooter	Total
17	7.92	0.00	0.00	6.24	0.00	2.84	0.00	0.14	17.13
23	10.76	0.06	0.18	0.54	22.73	0.35	5.05	0.00	39.68
24	0.29	0.00	0.00	1.78	0.00	0.00	0.00	0.00	2.06
25	0.71	0.00	0.00	9.47	0.00	0.00	0.75	0.00	10.92
29	29.88	0.00	0.00	4.60	0.92	39.32	0.00	0.18	74.9
37	0.00	0.00	0.00	28.13	0.00	0.00	0.00	0.00	28.13
39	1.27	0.00	0.00	1.47	0.82	3.78	0.00	0.00	7.35
40	6.22	0.02	0.00	0.27	5.66	0.00	2.27	0.00	14.43
18	6.59	3.31	9.41	15.8	0.00	0.00	0.00	5.98	41.09
20	0.02	0.00	0.00	0.00	0.00	0.00	0.14	0.00	0.15
26	0.03	0.00	0.00	0.12	0.00	0.00	0.00	0.00	0.14
27	1.87	0.00	0.00	0.83	0.00	0.78	0.00	0.00	3.47
28	0.04	0.00	0.00	0.00	0.00	0.00	0.00	0.00	0.04
31	0.16	0.00	0.00	0.29	0.39	0.00	0.00	0.00	0.85
35	0.09	0.00	0.00	2.07	0.00	0.00	0.00	0.00	2.16
**Total**	65.85	3.39	9.59	71.61	30.52	47.07	8.21	6.3	242.54

Table 3 presents the distribution of data collection per user for various transport modes in terms of distance traveled (km). The *Haversine* formula was employed to compute the geographical distance between two points in the trajectory, which calculates the shortest distance between two points on the surface of the Earth sphere [2]. The distance computation was based on the Global Positioning System (GPS) traces of users present in the original dataset, but the data is not published for the purpose of user anonymity. The distribution of data in terms of distance traveled has a significant impact on the distinction between motorized and non-motorized transport modes, as users of motorized transport modes cover greater distances at faster speeds.

**Table 3:** Distribution of data by transport mode per user expressed in km.

User ID	Walk	Run	Bike	Car	Bus	Train	Tram	E-scooter	Total
17	33.72	0.00	0.00	219.77	0.00	144.25	0.48	1.52	399.74
23	46.66	0.18	1.99	21.71	596.04	15.34	78.14	0.00	760.06
24	4.81	0.00	0.00	38.37	0.00	0.00	0.00	0.00	43.18
25	2.29	0.00	0.00	218.21	0.00	0.00	8.8	0.00	229.3
29	148.58	0.00	0.18	218.02	36.53	2077.94	0.00	1.47	2482.72
37	0.00	0.00	0.00	1797.05	0.00	0.00	0.00	0.00	1797.05
39	4.37	0.00	0.00	50.02	25.7	166.36	0.00	0.00	246.45
40	16.02	0.22	0.00	7.8	124.79	0.00	38.22	0.00	187.05
26	0.11	0.00	0.00	2.54	0.00	0.00	0.00	0.00	2.65
18	15.51	32.65	128.42	897.91	0.00	0.00	0.00	79.1	1153.59
28	0.02	0.00	0.00	0.00	0.00	0.00	0.00	0.00	0.02
27	3.72	0.00	0.00	25.6	0.00	29.72	0.00	0.00	59.04
31	0.26	0.00	0.00	10.22	9.9	0.00	0.00	0.00	20.38
35	7.44	0.00	0.00	30.15	0.00	0.00	0.00	0.00	37.59
20	0.07	0.00	0.00	0.00	0.00	0.00	1.52	0.00	1.59
**Total**	283.58	33.05	130.59	3537.37	792.96	2433.61	127.16	82.09	7420.41

Consequently, users who primarily use non-motorized transport modes cover shorter distances than those who primarily use motorized modes. For instance, when comparing users with IDs 18 and 37 in terms of time traveled, user 18 has more hours of collected data. However, the data collected from user 37, when expressed in terms of distance traveled, is approximately 60% greater than that of user 18.

**Fig. 2:**
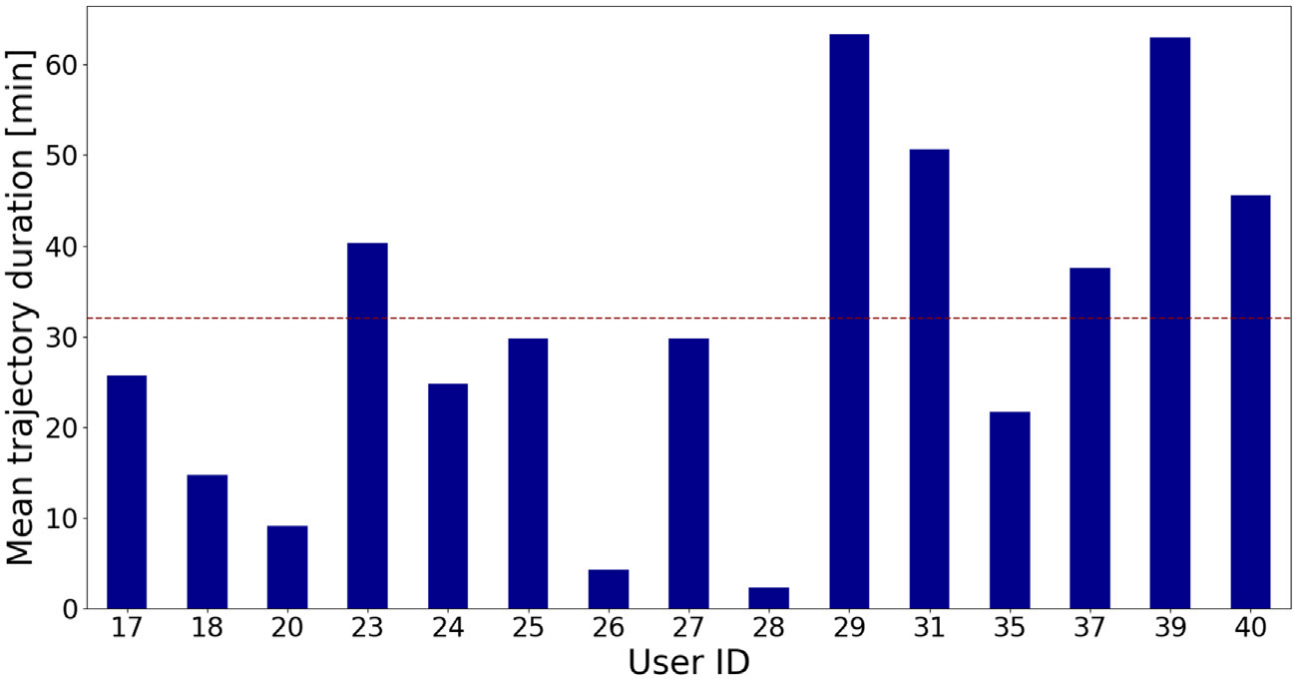
Average duration of user trajectories.

The duration of each trajectory varies and there are distinctions in trajectory duration among users, as illustrated in Fig. 2 which depicts the average trajectory duration for each user, with user IDs on the x-axis and average trajectory duration in minutes on the y-axis. The red dashed line indicates the average duration of trajectories for all users. The average trajectory duration for users 23, 29, 31, 37, 39, and 40 is higher than the average duration of all trajectories for the remaining users, whereas shorter trajectories are present for users 26 and 28.

**Fig. 3:**
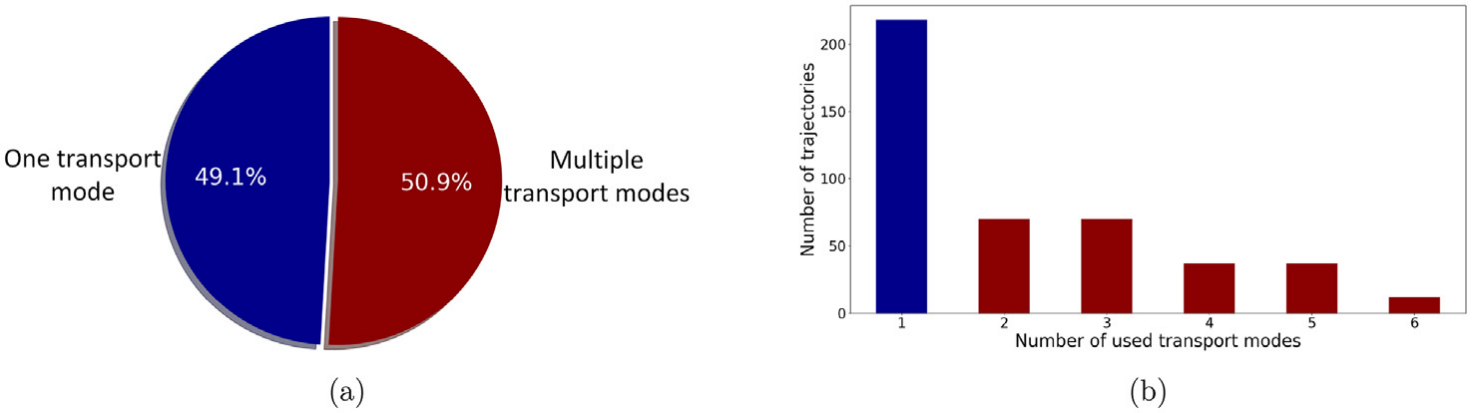
Distribution of the trajectory according to the number of used transport modes in the trajectory.

The trajectories can also be observed considering the number of transport modes used. Fig. 3 shows the distribution of trajectories according to the number of used transport modes. The proportion of trajectories with one or more transport modes is approximately equal, Fig. 3a. The distribution of the number of trajectories in which 1−6 transport modes were used is shown in Fig. 3b. For multimodal trajectories, the greatest number of trajectories consist of 2 and 3 transport modes, while the number of trajectories that include more transport modes is fewer.

## 4 Experimental Design, Materials and Methods

Data collection was conducted using a mobile application developed for devices with the Android operating system. Participants in the data collection were provided instructions on how to use the application upon installation on their personal mobile devices. During data collection, participants were prompted to:

1. activate the mobile application while navigating through the transportation network.
2. select the transport mode being used.
3. verify the accuracy of the acquired geographic data on the digital map as per the provided instructions.
4. deactivate the mobile application upon completion.

The proposed mobile application has been designed to function in a background mode, enabling concurrent use of other applications or screen lock on the device. A notification mechanism has been integrated to inform the user of the background activity. Users are given three key pieces of information about the transport mode classification: the time of the transport mode change, the standing classification, and the route summary, before being prompted for confirmation. Additionally, users are required to give their consent for data collection. To ensure users mark transport mode changes consistently, instructions are provided for users to make the transport mode change at the time of initiating use of the new transport mode. The second instruction pertains to standing, where users are instructed that standing is considered a part of walking (e.g., waiting for a bus). To validate the correctness of the trajectory, users should examine several segments of the trajectory. The first check involves the accuracy of the geographic positions of the displayed trajectory. The second check involves the verification of the transport modes used, i.e. whether the transport mode symbols on the digital map match the transport mode used. The final check pertains to the geographic locations of transport mode changes, where the user must confirm that the transport mode was actually changed at these locations.

**Fig. 4:**
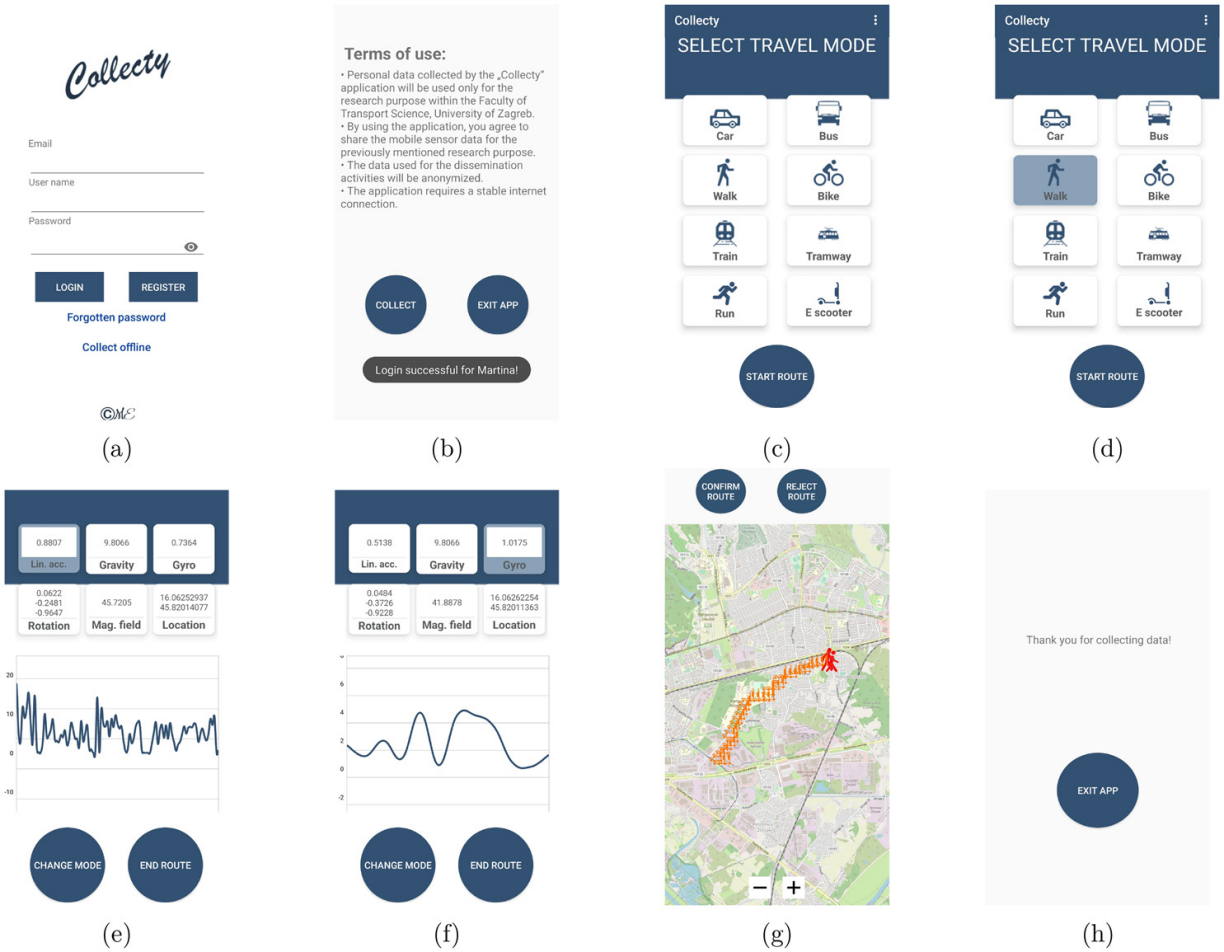
Android application Collecty used for data collection process.

The mobile application “*Collecty*” comprises of 6 consecutive activities, as shown in Fig. 4. The first activity pertains to user login or registration within the application, and the selection of data collection method (Fig. 4a). When the application is launched for the first time, registration within the system is mandatory. Two types of data collection are available: online and offline. The main distinction is that online data collection immediately sends all data from the mobile device to the server, while offline data collection stores sensor data in the mobile device’s internal memory. After logging in, the user can accept the terms of use for the application or exit the application if they do not agree with the terms (Fig. 4b). The second activity in the application is used for selecting the current transport mode (Fig. 4c), with 8 options available: Car, Bus, Walk, Bike, Train, Tram, Running, or Electric Scooter. The selected transport mode is highlighted in blue as shown in Fig. 4d. After selection by pressing the ”Start Route” button, data collection from mobile device sensors begins, and the selected transport mode is added to each record.

The third activity refers to monitoring the parameters from the sensors of the user’s mobile device and is used solely to visualize the data for the user. In the background, data are sent to the server or stored in the internal memory of the mobile device. As can be seen in the Fig. 4e and f, the selected sensor is highlighted in blue, and in the middle part the measured values of the selected sensor are displayed as a moving graph. Every second a new value is displayed in the graph. For the accelerometer, gravity, gyroscope and magnetometer, the values for magnitude are displayed. For the rotation, the values for all axes are displayed, and for the location, the longitude and latitude are displayed. In addition, this activity allows the user to change the transport mode (by pressing the *Change mode* button) or end the route (by pressing the *End route* button). When the user presses the button to change the transport mode, the application returns to the previous activity where the user can select the transport mode again. Another option is to complete the trajectory, which leads to the last activity. In the last activity, the user can verify the route on the digital map. The OpenStreetMap (OSM) was used to display the route, [3]. Fig. 4g shows the OSM interface with the user’s route plotted, with a part of the route marked with an icon corresponding to the transport mode used on that part of the route. The route where two transport modes were used is shown. Red pedestrian symbols mark the part of the route that the user walked, and the orange marking of the electric scooter marks the remaining part of the route. The user has two options: confirm the route (by pressing the *Confirm route* button) or reject the route (by pressing the *Reject route* button). Based on the user’s decision, all records of the displayed route will be classified as 1 (confirmed route), 2 (rejected route) or 3 (no decision made). GPS points are plotted on the digital map every 5 s or if more than 100 m have passed since the last GPS point. After user confirmation, a message is displayed and a button to exit the application (*Exit app*), as shown in Fig. 4h, appears.

## Ethics statements

Relevant informed consent was obtained from participants involved in data collection process and the participant data has been fully anonymized.

## CRediT authorship contribution statement

**Martina Erdelić:** Conceptualization, Data curation, Software, Validation, Formal analysis, Writing – original draft, Writing – review & editing. **Tomislav Erdelić:** Software, Data curation, Formal analysis, Writing – review & editing. **Tonči Carić:** Supervision, Writing – review & editing.

## Data Availability

Dataset for multimodal transport analytics (Original data) (Kaggle Data).
